# Beyond antibiotics: advances in photothermal strategies for oral infections

**DOI:** 10.3389/fbioe.2025.1637941

**Published:** 2025-11-12

**Authors:** Pei Wang, Jian Liang, Fen Liu

**Affiliations:** 1 School of Stomatology, Jiangxi Medical College, Nanchang University, Nanchang, Jiangxi, China; 2 Jiangxi Provincial Key Laboratory of Oral Diseases, Nanchang, Jiangxi, China; 3 Jiangxi Provincial Clinical Research Center for Oral Diseases, Nanchang, Jiangxi, China

**Keywords:** photothermal nanoparticles, biofilm disruption, antibiotic-free strategies, localized hyperthermia, oral immunotherapy

## Abstract

The rising prevalence of antibiotic resistance necessitates innovative alternatives for managing polymicrobial oral infections. Photothermal therapy (PTT) emerges as a revolutionary approach that transcends conventional antimicrobial limitations by leveraging near-infrared (NIR)-activated photothermal agents to generate localized hyperthermia, enabling precise biofilm eradication while circumventing systemic drug resistance. The modality capitalizes on the anatomical accessibility of oral tissues and the optical transparency of dental structures, allowing spatiotemporal control over pathogenic niches from superficial caries biofilms to deep periodontal pockets. Recent advances in nanoplatform engineering have unlocked multifunctional PTT systems capable of synergizing thermal ablation with immunomodulation, biofilm matrix penetration, and even tissue regeneration, addressing the dual challenges of microbial persistence and host inflammatory damage. However, clinical translation remains hindered by unresolved technical barriers, including optimal thermal dosage calibration, lesion-specific material design, and long-term biosafety assessment. This review systematically dissects cutting-edge photothermal strategies across the oral infectious spectrum (dental caries, endodontic infections, periodontitis, and peri-implantitis) while critically evaluating their mechanistic innovations in overcoming antibiotic limitations. We further propose a roadmap for next-generation smart PTT systems integrating stimulus-responsive materials and microbiome-aware therapeutic paradigms to achieve personalized oral infection management.

## 1 Introduction

As one of the most critical anatomical regions in the body, the oral cavity constitutes the initial segment of the digestive tract and maintains direct exposure to the external environment (Madani et al., 2014; Kunath et al., 2024). This unique anatomical and physiological positioning renders it highly susceptible to colonization by a diverse array of microorganisms. Structures such as teeth, gingival sulci, and mucosal surfaces provide a nutrient-rich ecological niche for these microbial communities to colonize, flourish, and thrive (Deo and Deshmukh, 2019; Brookes et al., 2023). The oral microbiome, recognized as the second most complex microbial ecosystem in the human body, predominantly colonizes the surface of oral mucosa and dentition (Kilian et al., 2016; Xiao et al., 2020; Baker et al., 2024). The maintenance of oral microbial homeostasis is critical for preserving both oral and systemic health. Multiple exogenous and endogenous factors—including dietary patterns, tobacco use, suboptimal oral hygiene practices, systemic comorbidities, and pharmacological interventions—can perturb the equilibrium of the oral microbiota, predisposing to pathogenic shifts (Sedghi et al., 2021; Gupta et al., 2024). Such dysbiosis states enable the proliferation of opportunistic pathogens, precipitating polymicrobial infections exemplified by periodontitis (Lamont et al., 2018; Jiang et al., 2021; Sedghi et al., 2021; Belibasakis et al., 2024). As a global health priority, oral infections rank among the most prevalent human infections, imposing significant socioeconomic burdens on healthcare infrastructure and international economies (Peres et al., 2019; Bernabe et al., 2020; Collaborators, 2025; Zheng et al., 2025). Clinically, these diseases often present with symptoms such as toothache and gingival inflammation, which can significantly impair mastication, communication, and aesthetic function, ultimately diminishing people’s quality of life (Spanemberg et al., 2019; Popescu et al., 2024). Moreover, emerging evidence underscores a compelling association between oral infections and an elevated risk of systemic disorders, including diabetes mellitus, atherosclerosis, and Alzheimer’s disease (Scannapieco and Cantos, 2016; Altamura et al., 2024; Popescu et al., 2024; Villoria et al., 2024). Consequently, the prevention, management, and therapeutic intervention of oral infections have garnered considerable scientific and clinical attention, underscoring the imperative for interdisciplinary research and innovative strategies to mitigate their global impact.

Oral infections comprise a diverse group of highly prevalent conditions, including dental caries, endodontics, periodontitis, and peri-implantitis, etc (Gondivkar et al., 2019; Peres et al., 2019). The management and treatment of these diseases are characterized by three distinct features: First, the affected organs (such as the pulp chamber, root canal, and periodontal tissues) are small in volume yet anatomically complex, making it difficult to completely eradicate infections, which often results in suboptimal treatment outcomes or even failure. Second, most of the oral infections are oral biofilm infection-associated diseases (Muras et al., 2022; Pan et al., 2025). Extracellular polymeric substances (EPS) in microbial biofilms offer adhesion and protection, rendering innate immune cells and conventional antimicrobials ineffective at breaking down oral biofilms and eradicating the microbes they contain (Bowen et al., 2018; Chen et al., 2023a). Third, the oral and maxillofacial region, being crucial for speech, mastication, respiration, and aesthetics, possesses complex physiological and psychological functions, necessitating minimally invasive treatment approaches that preserve function. Oral infections typically necessitate the removal of pathogenic bacteria and their biofilms (Ertem et al., 2017; Pitts and Mayne, 2021; Alawaji et al., 2022). Current clinical approaches are based on mechanical removal supplemented by antibiotics, such as scaling and root planning (SRP) therapy combined with minocycline for periodontitis (Mombelli, 2018; Sanz et al., 2020; Laforgia et al., 2024). However, the effectiveness of the traditional mechanical bacteria and biofilms removal method is primarily compromised by the intricate and small anatomical structures of the organs (Li et al., 2021; Lin et al., 2024). Meanwhile, inappropriate antibiotic use has led to bacterial resistance, including multidrug-resistant bacteria (Hernando-Amado et al., 2019; Rams et al., 2020). Oral biofilms significantly contribute to drug resistance, as their matrix effectively bars the penetration and activation of antibiotics. Moreover, as bacteria expand and metabolic residues accumulate, the resulting acidic shift within the biofilm environment not only inactivates antibiotics but also compromises their overall effect. Therefore, alternative non-antibiotic-dependent antimicrobial strategies are required to address these issues.

**FIGURE 1:**
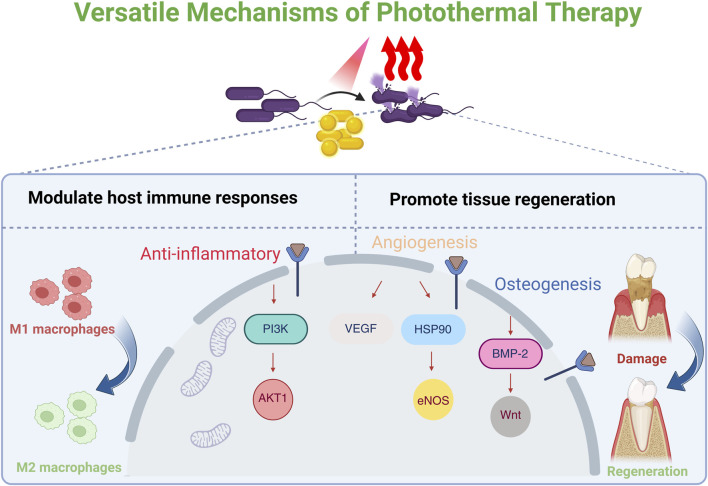
Schematic illustration of the versatile mechanisms of photothermal therapy (PTT). PTT could modulate the host’s biological response. This includes promoting the polarization of pro-inflammatory M1 macrophages towards an anti-inflammatory M2 phenotype via the PI3K-AKT1 signaling pathway. The resulting pro-regenerative microenvironment enhances angiogenesis through the VEGF/HSP90/eNOS pathway and promotes osteogenesis by activating the BMP-2 and Wnt signaling pathways. Created in BioRender. Liang, J. (2025) https://BioRender.com/6vgs15f.

Widely utilized across various fields, including antimicrobial applications, photothermal therapy (PTT) represents a promising strategy for the treatment of oral infections (He et al., 2023; Wang et al., 2024; Liang et al., 2025; Zhang and Chen, 2025). PTT operates by exposing photothermal agents (PTAs) to light at a specific wavelength (e.g., visible or near-infrared, NIR), which facilitates the interaction of photons with the PTAs’ surface (Overchuk et al., 2023). This interaction induces molecular vibrations and rotations, converting the absorbed energy into heat and elevating the local temperature (Fang et al., 2025; Zhang et al., 2025). Elevated temperatures disrupt bacterial cell membranes, compromising their structural integrity and increasing permeability, which leads to the leakage of essential intracellular components (Cao et al., 2024; Mondal et al., 2024). Concurrently, crucial bacterial proteins involved in replication, metabolic processes, and survival are denatured, ultimately leading to bacterial demise (Yin et al., 2019). Unlike antibiotics, PTT’s physical antibacterial mechanism offers broad-spectrum capabilities, a low likelihood of inducing drug resistance, and the ability to circumvent pre-existing drug-resistant bacterial strains (Cao et al., 2024). PTT has also demonstrated significant advantages in combating biofilms. PTAs, especially in nanoparticle form, can readily traverse the EPS to access the embedded microbial cells (Pinto et al., 2020). Moreover, hyperthermia exhibits significant potential to disrupt the intrinsic physiological microenvironment of biofilms by inactivating their inherently bioactive substrates, such as nucleic acids and proteins, which contribute to the degradation of oral biofilms (Liu et al., 2021; Chen et al., 2023b; Mammari and Duval, 2023). It has also been reported that PTT can disrupt pathogen co-aggregation via the Cbe-Ltp1-Ptk1-fimA signaling pathway, thereby preventing biofilm development (Lin et al., 2024). While high temperatures (>50 °C) inhibit bacterial growth, mild PTT (mPTT; <45 °C) can modulate host immune responses and promote tissue regeneration (Sheng et al., 2021; Zhang et al., 2021; Huang et al., 2022; Li et al., 2022; Xue et al., 2022; Xue et al., 2023). Extensive studies have demonstrated that periodic mild PTT, by maintaining local temperatures at approximately 40 °C–43 °C for short durations (e.g., 3–5 min) repeated several times, can substantially mitigate inflammation and accelerate both angiogenesis and osteogenesis (Zhang et al., 2019; Li et al., 2022; Wu et al., 2022; Zeng et al., 2023; You et al., 2024). Thermal stimulation could regulate macrophage polarization by activating the PI3K-AKT1 signaling pathway, which promotes the phenotypic transition of pro-inflammatory M1 macrophages towards an anti-inflammatory and pro-reparative M2 state. Angiogenesis might be fostered through the vascular endothelial growth factor (VEGF), heat shock protein 90 (HSP90)/endothelial nitric oxide synthase (eNOS) pathways, while osteogenesis might be promoted by enhancing bone morphogenetic protein-2 (BMP-2) expression and activating the Wnt signaling pathway (Zhang et al., 2019; Sheng et al., 2021). Therefore, PTT offers a powerful and versatile approach, integrating potent antimicrobial activity with desirable anti-inflammatory and pro-regenerative functions, making it a valuable strategy when combined with other antimicrobial and regenerative strategies (Chen et al., 2023b). As a non-antibiotic-dependent antimicrobial strategy, PTT offers antibacterial performance superior to that of antibiotics. This advantage stems from its non-invasive, spatiotemporal, and site-selective characteristics, strong tissue penetration, low side effects, broad-spectrum antibacterial properties, inherent resistance to the development of drug resistance, and versatile nature as a therapeutic platform (Wu J. et al., 2019) (Figure 1).

**FIGURE 2:**
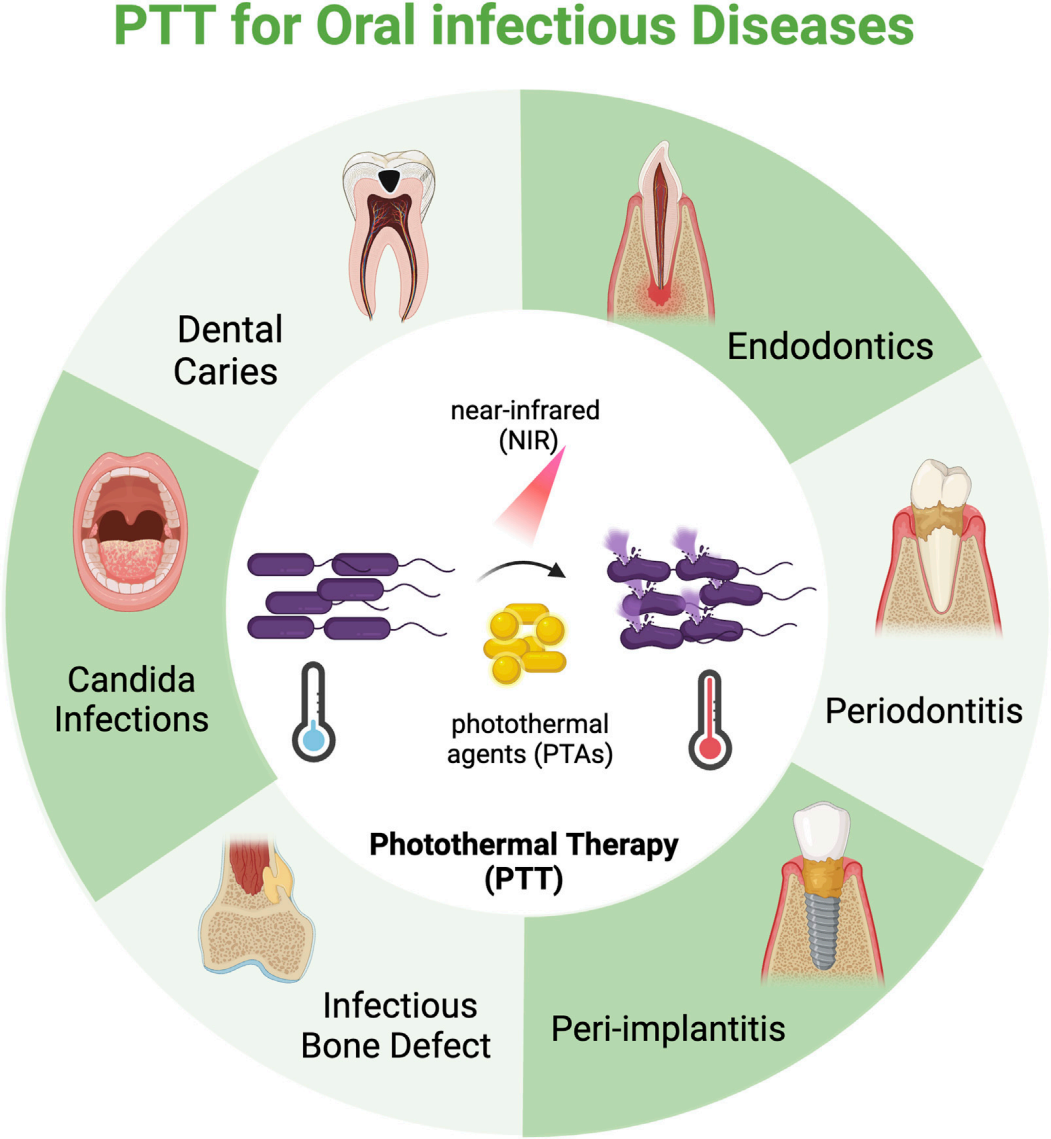
Schematic illustration of photothermal therapy (PTT) for oral infectious diseases. By leveraging photothermal agents (PTAs), PTT converts near-infrared (NIR) laser into localized hyperthermia to induce pathogen mortality. The application of PTT to oral infectious diseases has predominantly seen progress in the areas of dental caries, endodontics, periodontics, and peri-implantitis, but also holds significant promise for treating other infections like infectious bone defects and *candida* infections. Created in BioRender. Liang, J. (2025) https://BioRender.com/6vgs15f.

The efficacy of PTT for oral infections hinges on two factors: the PTAs and the light sources. While the superficial anatomical location of oral tissue mitigates concerns regarding penetration depth, PTT’s overall effectiveness is primarily limited by the photothermal conversion efficiency (PCE) of PTAs and the risk of collateral thermal damage to healthy tissues from overheating (Liu et al., 2019; Yu S. et al., 2024). Over the past few decades, advancements in nanomaterials have significantly improved the PCE of PTAs (Zhao et al., 2024). Research has gradually shifted from focusing solely on the photothermal properties of individual materials to strategically designing and synthesizing multifunctional platforms. These platforms integrate features like targeted drug delivery, synergistic antibacterial action, and combinational immune modulation, thereby enhancing therapeutic efficacy while minimizing adverse effects. This field has rapidly progressed, yielding encouraging results. While existing reviews primarily focus on specific materials [e.g., gold-based nanomaterials (Zhang S. et al., 2024; Qi et al., 2025)] or single diseases [e.g., dental caries (Xu et al., 2025) and periodontitis (Li J. et al., 2024)], a comprehensive, interdisciplinary overview of the broader spectrum of major oral infections is still missing. Here, we summarize the advances of PTT in oral infections with a focus on dental caries, endodontics, periodontics, and peri-implantitis, highlight the design concepts and mechanisms, address the challenges PTT faces, and suggest future directions (Figure 2). We aim to provide a foundational framework for advancing PTT research for the treatment of oral infections and to catalyze the development of precise, efficient, and clinically viable therapeutic strategies (Table 1).

**TABLE 1:** Summaries of photothermal therapy (PTT) for oral infectious diseases. This section comprehensively summarizes the details of recent research reported in the literature, emphasizing the functionalization of photothermal agents (PTAs), their therapeutic highlights, and the underlying design concepts and mechanisms.

Disease type	Therapy model	Photothermal agents	Functionalization	Exposure condition	Bacterial species	Research phase	Therapeutic highlights	Ref.
Dental Caries	PTT	PDA	Fe_3_O_4_ NPs loaded with Ag via PDA reduction and grafted with glycol chitosan post-PDA coating	808 nm, 0.75 W cm^-2^, 10 min	*S. mutans*	Planktonic bacteria, biofilms	Ag-enhanced PTT, pH-responsive release of Ag^+^, magnetically retrievable nano agents	Xu et al. (2020a)
	PTT, drug therapy		ZIF-8 coated with PDA	808 nm, 1.5 W cm^-2^, 10 min	*S. mutans*	Planktonic bacteria, biofilms, animal model	Synergistic oral biofilm eradication using pH-responsive Zn^2+^ release and photothermal effect	Pan et al. (2025)
	PTT, PDT, drug therapy	IR780	Poly (ethylene glycol)-b-poly(3-acrylamide phenylboronic acid)-b-poly(2-(5,5-dimethyl-1,3-dioxan-2-yloxy) ethyl acrylate) dual block copolymers co-encapsulating ciprofloxacin and IR780	808 nm, 1.5 W cm^-2^, 5 min	*S. mutans*	Planktonic bacteria, biofilms, isolated dental model, animal model	Integrating biofilm penetration and bacterial anchoring for targeted drug delivery	Yu et al. (2022)
	PTT, Drug Therapy	GO	Amino-functionalized	808 nm, 0.88 W cm^-2^, 5 min	*S. mutans*	Planktonic bacteria	Amino-GO with integrated positive charge, strong photothermal effect, and inherent cutting effect	Lu et al. (2021)
	PTT	PB NPs	Ag^+^-doped Prussian blue nanoparticles encased in cationic guar gum	808 nm, 0.4 W cm^-2^, 3 min	*S. mutans*, *S. sobrinus*, *S. sanguinis*	Planktonic bacteria, biofilms, animal model	Combined PTT with Ag^+^ release for enhanced and safer caries treatment	Li et al. (2024b)
	PTT, PDT	Zinc phthalocyanine tetrasulfonate (ZnPcS_4_)	ZnPcS_4_ with surface modification by guanidinium-functionalized, fluorocarbon-grafted calix [5]arene	660 nm, 1 W cm^-2^, 5 min	*S. mutans*	Planktonic bacteria, biofilms, *in vitro* human biofilms model, animal model	Adaptive PTT and PDT enhancement enabling on-demand modality switching	Zhang et al. (2024b)
	PTT	BP NSs	Encapsulated in chitosan and PLGA-PEG-PLGA hydrogel matrices	808 nm, 1 W cm^-2^, 5 min	*S. mutans*, *S. sanguinis*	Planktonic bacteria, animal model	Highly efficient bactericidal and remineralization-promoting effects	Ran et al. (2024)
Endodontics	PTT	AuNRs	—	810 nm, 0.2 W, 20 min	*E. faecalis*	*In vitro* biofilms	Mature *E. faecalis* biofilm developed in roots using a Modified Drip Flow Reactor (MDFR) and a Static Method	Galdámez-Falla et al. (2022)
	PTT, PCT	AuNPs	AuNPs integrated onto Cu_2-x_S	808 nm, 0.5 W cm^-2^, 10 min	*E. faecalis*, *F. nucleatum*	Isolated dental models, animal model	Combining PTT and peroxidase-like catalytic therapy (PCT) to enhance biofilm bacteria eradication in root canals	Cao et al. (2021)
	PTT	AuAg core-shell	—	808 nm, 1 W cm^-2^, 10 min	*E. faecalis*	Planktonic bacteria, biofilms	Effective antibacterial agents against Ag^+^-resistant *E. faecalis*	Feng et al. (2024)
	PTT, PDT	BP NSs	BP NSs decorated with monodisperse AuNPs	808 nm, 1 W cm^-2^, 5 min	*E. faecalis*	Planktonic bacteria, biofilms	First study on antibacterial and antibiofilm activity of BP/Au nanocomposites via NIR light-mediated photothermal process	Aksoy et al. (2020)
	PTT, chemotherapy	Two isoindigo (DIID)-based semiconducting conjugated polymer (PBDT-DIID)	PBDT-DIID NP core incorporating polylactide	808 nm, 0.8 W cm^-2^, 2.5 min	*E. faecalis*	Isolated dental model	Photothermal enhancement of root canal treatment outcome by heating 1% NaClO solution	Duan et al. (2022)
Periodontitis	PTT, CDT	PDA	Cu_2_O NPs and PDA-coated titanium dioxide loaded within a hydrogel composite	NIR, 1.00 W cm^-2^, 18 min 452 nm, 5.52 W m^-2^, 5 min	*S. aureus*, *E. coli*, *S. mutans*	Planktonic bacteria, animal model	ROS generation boosts antibacterial efficacy and facilitates Cu^+^ oxidation to Cu^2+^, synergistically promoting osteogenesis with the photothermal effect	Xu et al. (2020b)
	PTT, immunotherapy	AuAg NPs	Branched AuAg NPs with a procyanidin-Fe network surface loading	808 nm, 2.5 W cm^-2^, 3 min	*P. gingivalis*, *F. nucleatum*	Planktonic bacteria, animal model	ROS scavenging and promotion of M2 macrophage polarization via the PI3K/AKT pathway, leading to immunity regulation	Wang et al. (2022a2)
	PTT, immunotherapy		AuAg NPs loaded with procyanidins	808 nm, 2.5 W cm^-2^, 5 min	*P. gingivalis*	Planktonic bacteria, animal model	Ag^+^ enhanced PTT provides antibacterial effect, while procyanidins regulate host immunity by scavenging ROS, inhibiting inflammation, and modulating macrophage polarization	Wang et al. (2023a)
	PTT, CDT	CuS NPs	CuS and MnS co-crystallized into nanosheets, enabling MnO_2_ layer-assisted synthesis of CuS/MnS@MnO_2_	808 nm, 1 W cm^-2^, 5 min	*P. gingivalis*, *F. nucleatum*	Planktonic bacteria, biofilms, animal model	Single nanocrystalline material achieving PTT and CDT for maximized nanomedicine synergy	Chen et al. (2023c)
	PTT		CuS NPs precipitated with chitosan, then methacrylated and photo-crosslinked with GelMA to form hybrid hydrogels	808 nm, 1 W cm^-2^, 5 min	*E. coli*, *S. aureus*, MRSA	Planktonic bacteria, animal model	Injectable hybrid hydrogels achieved both enhanced osteogenesis and NIR-triggered sterilization	Yang et al. (2025)
	PTT, PDT		CuS loaded with serine endopeptidase	980 nm, 1.5 W cm^-2^, 3 min	*F. nucleatum*	Planktonic bacteria, biofilms, animal model	Enzymatic degradation of the biofilm by introducing a protease	Gao et al. (2023)
	PTT, drug therapy	GNC	GNR filled with phase-change materials (PCM) and tetracycline (TC), with a surface modification of poly(N-isopropylacrylamide-co-diethylaminoethyl methacrylate) (PND)	808 nm, 1.0 W cm^-2^, 3 min	*S. aureus*	Planktonic bacteria, animal model	Precise NIR light-controlled release of encapsulated drugs via dual thermosensitive transitions of PCM (liquid-solid) and PND (coil-granule)	Zhang et al. (2020)
	PTT, drug therapy	Au nano bipyramids	Mesoporous silica-coated Au nano bipyramids mixed with gelatin methacrylate	808 nm, 1.2 W cm^-2^, 5 min	*P. gingivalis*	Planktonic bacteria	Antibiotic drug release and photothermal treatment triggered by NIR irradiation	Lin et al. (2020)
	PTT, PDT	ICG	ICG complexed with sPDMA, a poly(2-(dimethylamino)ethyl methacrylate) brush synthesized by ATRP using bromo-β-cyclodextrin (CD-Br) initiator	808 nm, 2 W cm^-2^, 5 min	*P. gingivalis*	Planktonic bacteria, animal model	Polycationic brushes as a novel carrier material for antibacterial agents	Shi et al. (2021)
	PTT, SRP		—	810 nm, 0.5 W, 1.5 min	-	Clinical randomized controlled trial	Evaluation of ICG-diode laser effects on periodontal cells with regenerative capacity	Chiang et al. (2020)
	PTT, immunotherapy	MPB NPs	MPB NPs loaded with baicalein	808 nm, 1 W cm^-2^, 15 min	*P. gingivalis*, *F. nucleatum*	Planktonic bacteria, animal model	ROS-scavenging nanoplatform promotes M2 macrophage polarization via photothermal bioplatform-assisted immunotherapy	Tian et al. (2022)
	PTT, PDT, immunotherapy	AuNRs	S-nitrosothiols and ICG loaded into mesoporous silica-coated AuNRs	808 nm, 1 W cm^-2^, 5 min	*P. gingivalis*, *F. nucleatum*	Planktonic bacteria, biofilms, animal model	NIR light triggers antibacterial effects (AuNR, PTT), anti-inflammatory action (ICG, PDT), and modulation of inflammatory immunity (generated NO)	Qi et al. (2022)
	PTT, Drug therapy, immunotherapy	PB NPs	PB NPs coated with PDA and subsequently loaded with minocycline	808 nm, 1 W cm^-2^, 5 min	*S. sanguinis*, *P. gingivalis*, *F. nucleatum*	Planktonic bacteria, biofilms, animal model	Mild temperature anti-plaque activity and ROS scavenging attributed to PB nanozymes (enzyme-like activity) and PDA (catechol reducibility)	Wang et al. (2023b)
	PTT, PDT	IR820	IR820 complexed with oxyhemoglobin	808 nm, 2 W cm^-2^, 5 min	*P. gingivalis*	*In vitro* biofilms, animal model	Hemoglobin as a carrier for targeted delivery of therapeutics to *P. gingivalis*	Bai et al. (2022)
	PTT, CDT	Cu_3_P	Cu_3_P modified with poly (allylamine hydrochloride) and lactate oxidase	1064 nm, 0.75 W cm^-2^, 5 min	*S. gordonii*, *P. gingivalis*	*In vitro* biofilms, animal model	Single-material system with PTT and CDT functionalities exhibiting synergistic therapeutic efficiency through a dynamic positive feedback loop	Lin et al. (2024)
	PTT, PDT	T8IC NPs	Hydrogel with 3D network architecture as a carrier for BMP-2 and T8IC	808 nm, 1.5 W cm^-2^, 5 min	*P. gingivalis*	Planktonic bacteria, biofilms, animal model	Enhanced PDT and sustained BMP-2 release achieved with mild PTT (45 °C) in a Hydrogel + T8IC + Laser + BMP-2 + H_2_O_2_ system, demonstrating excellent bactericidal effect, osteogenic induction, and biosafety	Wang et al. (2023c)
	PTT, PDT, CDT	Bi_2_S_3_ NPs	Bi_2_S_3_ NPs anchored on Cu-tetrakis(4-carboxyphenyl)porphyrin nanosheets to create a novel Z-scheme heterostructured nanocomposite	635 nm,1 W cm^-2^, 10 min	*P. gingivalis*, *F. nucleatum*, *S. gordonii*	Planktonic bacteria, biofilms, animal model	Heterostructure facilitates highly efficient light absorption and electron-hole separation, leading to synergistic PDT/PTT/CDT with potent antibacterial activity against periodontal pathogens	Kong et al. (2023)
	PTT, drug therapy	Fe_3_O_4_	Fe_3_O_4_ wrapped ZnO with an outer layer of epsilon-polylysine (EPL)	808 nm, 1 W cm^-2^, 5 min	*P. gingivalis*	Planktonic bacteria, biofilms, animal model	Anti-inflammatory effects and enhanced antibiofilm efficacy via mild-temperature antibacterial PTT	Li et al. (2025)
	PTT, CDT	Bi_2_Te_3_ NSs	Lu-Bi_2_Te_3_ decorated with Fe_3_O_4_ and poly(ethylene glycol)-b-poly(l-arginine) (PEG-b-PArg)	1064 nm, 1 W cm^-2^, 5 min	*P. gingivalis*, *F. nucleatum*, *S. aureus*, *E. coli*	Planktonic bacteria, biofilms, animal model	Synergistic generation of ROS and RNS via photothermal/thermocatalytic effects under NIR-II laser irradiation leads to biofilm damage	Dai et al. (2023)
	PTT, gas therapy	PB nanozymes	Ruthenium (Ru)-doped PB nanozymes integrated with sodium nitroprusside (SNP)	808 nm, 1 W cm^-2^, 5 min	*P. gingivalis*, *F. nucleatum*	Planktonic bacteria, biofilms, animal model	NO-releasing nanozyme therapy using mild-temperature photothermal activation	Li et al. (2024e)
	PTT, PDT, gas therapy	Ag_2_S	Ag_2_S NPs loaded with ZIF-90, ICG, and L-arg molecule	808 nm, 1 W cm^-2^, 5 min	*P. gingivalis*, *F. nucleatum*	Planktonic bacteria, biofilms, animal model	NO-synergized PTT and PDT using a nanocomposite platform	Wu et al. (2023)
	PTT, immunotherapy	AuNPs	Yolk–Shell structure composed of Au and CeO_2_ loaded with dimethyl fumarate	635 nm, 0.8 W cm^-2^, 5 min	*E. coli*, *S. aureus*	Planktonic bacteria, animal model	Triple-combination therapy for periodontitis enabled through antioxidant, mitochondrial maintenance, and immunomodulation	Li et al. (2024d)
Peri-implantitis	PTT	TiO_2_	(Si/P/F) multi-doped porous TiO2 matrix	808 nm, 0.6 W cm^-2^, 5 min	*S. aureus*	Planktonic bacteria, biofilms, animal model	Endowing dental implants with superior bactericidal ability, accelerated epithelial sealing and osseointegration, and reduced alveolar resorption	Xue et al. (2023)
	PTT	ICG	—	810 nm, 0.67 W cm^-2^, Not mentioned	*S. gordonii*	*In vitro* biofilms	First evaluation of the antimicrobial effect of PTT on zirconia surfaces	Shim et al. (2022)
	PTT, PDT		ICG and rapamycin encapsulated within liposomes	808 nm, 1.5 W cm^-2^, 5 min	*S. aureus*, *S. oralis*	Planktonic bacteria, biofilms, animal model	Increases bacterial motility by elevating intracellular ATP, inhibits bacterial adhesion and biofilm formation, thus preventing disease recurrence	Xiao et al. (2024)
	PTT	GO	Reduced GO (rGO)	940 nm, 4 W cm^-2^, 2 min	*S. mutans*, *P. gingivalis*	Planktonic bacteria	Zirconia coated with rGO via atmospheric plasma to eliminate implant surface plaque	Park et al. (2023)
	PTT	PDA	—	808 nm, 1 W cm^-2^, 5 min	*S. aureus*	Planktonic bacteria, *In vitro* 3D peri-implantitis model	First report of collateral thermal damage to tissues overlying an implant surface coated with photothermal NPs	Ren et al. (2020)
	PTT		Simvastatin-loaded ZIF-8 nanoparticles coated with PDA and subsequently incorporated into a Chitosan (CS)/β-glycerophosphate (β-GP) system	808 nm, 0.5 W cm^-2^, 10 min	*S. aureus*, *P. gingivalis*	Planktonic bacteria, biofilms, animal model	Demonstrated attenuation of infection and inflammation in peri-implantitis lesions	Liu et al. (2025)
	PTT, PDT		Ce6-loaded ZIF-8 nanoparticles coated with PDA/UBI	660 nm, 1.3 W cm^-2^, 5 min	*S. aureus*, *E. coli*	Planktonic bacteria, biofilms, animal model	Precise targeting of bacteria and enhanced oral biofilm penetration	Wang et al. (2025)
Infectious bone defect	PTT	MXene	MXene (Ti_3_C_2_) incorporated into a 3D bioprinted composite hydrogel scaffold composed of GelMA, β-TCP, and Sodium alginate (Sr^2+^)	808 nm, 1.5 W cm^-2^, 5 min	*S. aureus*, *E. coli*	Planktonic bacteria, animal model	Personalized bone tissue engineering scaffolds exhibiting synergistic antibacterial and osteogenic effects	Nie et al. (2022)
	PTT	MgMps	MgMps combined with PLLA to form a lamellar heterostructured Mg/PLLA composite membrane via accumulative rolling	808 nm, 0.7 W cm^-2^, 1 min	*E. coli*, *S. aureus*	Planktonic bacteria, animal model	Programmed degradation to release Mg^2+^, antibacterial efficacy and endogenous vascularized bone regeneration ability	Wang et al. (2023d)
*Candida* infections	PTT	MPN-Pd	Metal-phenolic networks with Pd nanoparticle nodes (MPN-Pd)	808 nm, 1 W cm^-2^, 45 min	*C. albicans*	Planktonic bacteria, biofilms, animal model	Demonstrated PTT’s potential against oral fungus infection	Chen et al. (2023a)

Abbreviation. 3D, three-dimensional; ATP, adenosine-triphosphate; ATRP, atom transfer radical polymerization; AuNPs, gold nanoparticles; AuNRs, gold nanorods; BMP-2, bone morphogenetic protein-2; BP, black phosphorus; *C. albicans, Candida albicans*; CDT, chemical dynamic therapy; *E. coli, Escherichia coli*; *E. faecalis: Enterococcus faecalis*; EPL: epsilon-polylysine; *F. nucleatum: Fusobacterium nucleatum*; GelMA, gelatin methacrylate; GNC, gold nanocages; GO, graphene oxide; ICG, indocyanine green; MgMps, Mg microparticles; MPB, mesoporous Prussian blue; MPN, metal-phenolic networks; MRSA, Methicillin-resistant *Staphylococcus aureus*; NIR, near-infrared; NPs, nanoparticles; NSs: nanosheets; *P. gingivalis: Porphyromonas gingivalis*; PB, prussian blue; PCM, phase-change materials; PCT, peroxidase-like catalytic therapy; PDA, polydopamine; PDT, photodynamic therapy; PEG, polyethylene glycol; PLLA, polylactic acid; PLGA, poly lactic acid-co-glycolic acid; PTT, photothermal therapy; rGO, reduced graphene oxide; RNS, reactive nitrogen species; ROS: reactive oxygen species; *S. aureus, Staphylococcus aureus*; *S. gordonii: Streptococcus gordonii; S. mutans, Streptococcus mutans;* SNP, sodium nitroprusside; *S. oralis, Streptococcus oralis; S. sanguinis, Streptococcus sanguinis; S. sobrinus, Streptococcus sobrinus*; SRP, scaling and root planning; TC, tetracycline; UBI, ubiquicidine; ZIF, zinc imidazolate framework.

## 2 PTT for dental caries

Dental caries is a chronic condition precipitated by the accumulation of dental plaque, metabolic acid production, and subsequent localized demineralization of hard tissues, which can lead to severe tooth defects (Pitts et al., 2017; Shen et al., 2024; Zhao et al., 2024). Dental plaque, composed of cariogenic microbial biofilms, serves as a critical etiological driver in dental caries development, with *Streptococcus mutans* (*S. mutans*) being the primary cariogenic pathogen (Li et al., 2024d; Mazurel et al., 2025). The primary approach to preventing and treating caries involves using antimicrobial drugs combined with mechanical removal of decayed tissue (Akindele et al., 2025; Song et al., 2025). However, biofilms impede the penetration of antimicrobial drugs and enable bacteria to adapt their metabolic states to the biofilm microenvironment, thereby fostering antibiotic resistance (Jakubovics et al., 2021; Hajishengallis et al., 2023). Moreover, mechanical removal often damages healthy tooth structures and is prone to caries recurrence (AlSahafi et al., 2022). There have been innovative approaches for caries prevention and treatment, including therapeutics to prevent the demineralization caused by dental biofilm (Li et al., 2024d) (novel chemoprophylactic agents, antimicrobial peptides, probiotics and replacement therapy, etc.) as well as therapeutics to promote the remineralization process (fluoride and casein phosphopeptides, etc.) (Chen and Wang, 2010). However, the implementation of these methods is limited by factors such as mucosal irritation, systemic toxicity, the development of drug-resistant microbes, and the inability to maintain adequate drug concentrations in the oral cavity.

**FIGURE 3:**
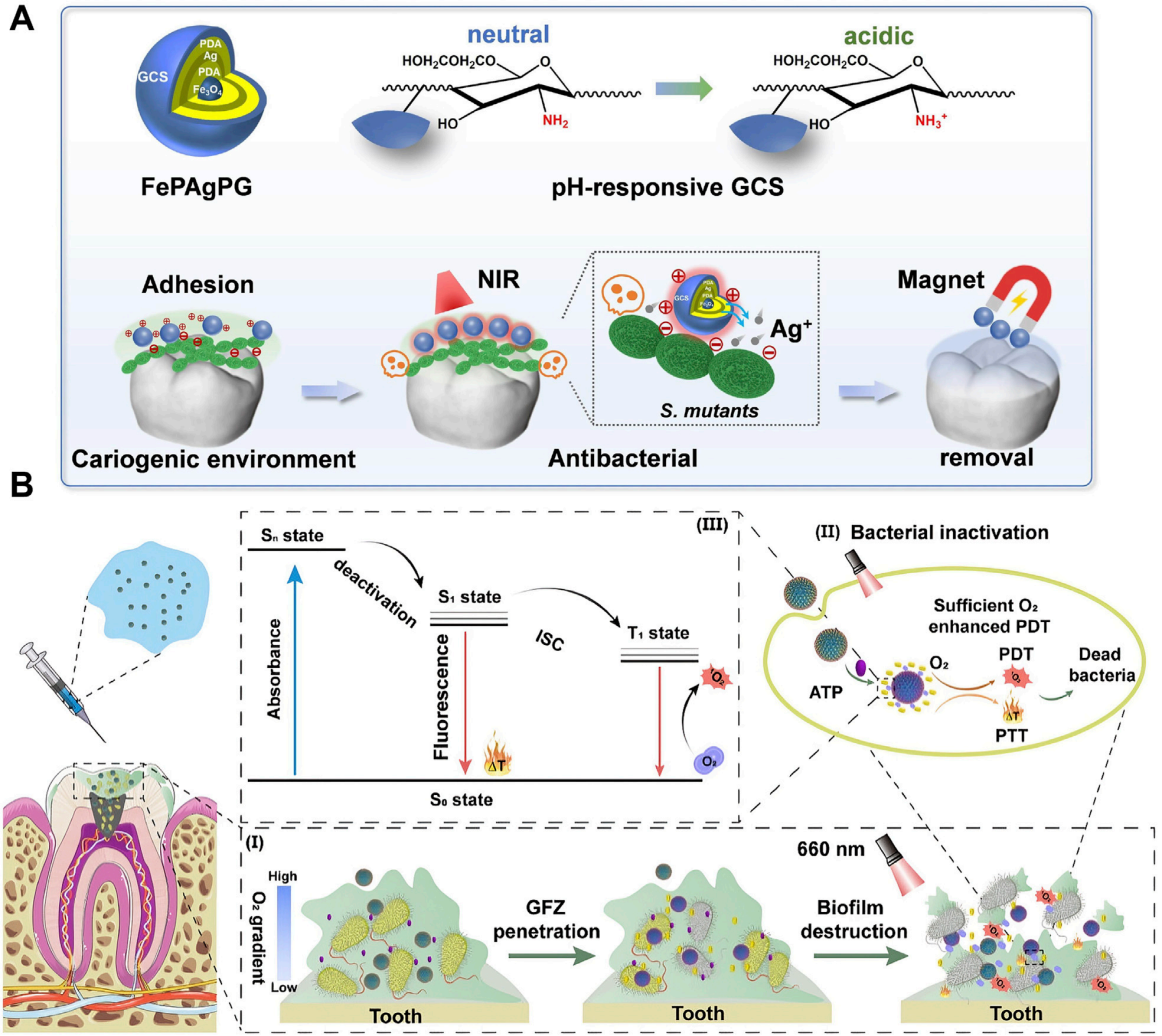
Photothermal therapy (PTT) for dental caries. **(A)** Schematic illustration depicting a removable photothermal antibacterial “warm paste” designed to target cariogenic bacteria. Reproduced with permission (Xu H. et al., 2022). Copyright 2021, Elsevier Group. **(B)** Schematic illustration of effective biofilm removal using a supramolecular nanoformulation featuring adaptive photothermal/photodynamic conversion. Reproduced with permission (Zhang Y. et al., 2024). Copyright 2024, American Chemical Society.

The unique properties of PTT, such as its ability to deliver localized and controlled thermal energy, make it a promising alternative to conventional methods for managing dental caries. By leveraging the photothermal effect, PTT can selectively target cariogenic biofilms without causing significant damage to surrounding healthy tissues (Zhu et al., 2014; Tan et al., 2018; Yang et al., 2018). Since cariogenic bacteria generate an acidic microenvironment through biofilm formation and acid production on tooth surfaces, pH-responsive targeting strategies have been developed for PTAs (Xu H. et al., 2022; Yu et al., 2022). A photothermal antibacterial “warm paste” was fabricated by loading Ag onto the surface of Fe_3_O_4_ nanoparticles by polydopamine (PDA) reduction, followed by a second PDA coating and subsequent grafting with glycol chitosan. Under normal physiological conditions, the PDA layer inhibits the excessive release of Ag^+^ and reduces its damage to normal tissues. However, within a cariogenic acidic environment, the protonation of amine groups on glycol chitosan leads to a positive charge on the nanoparticles, which enhances their strong adhesion to negatively charged cariogenic bacteria at the intended site. When irradiated by NIR, the increased temperature promotes Ag + release, leading to a high local concentration in the cavitated dental tissue. This thereby achieves effective targeted antimicrobial action through an Ag-assisted PTT strategy (Figure 3A) (Xu H. et al., 2022). Other pH-responsive agents tailored for acidic oral niches include zinc imidazolate framework-8 (ZIF-8) and Poly (ethylene glycol) (PEG) etc (Yu et al., 2022; Pan et al., 2025). The positive charge of some modified PTAs like amino-functionalized graphene oxide (GO) ensures their strong interaction with the negatively charged bacterial cells, which can also be helpful for the target of cariogenic bacteria (Lu et al., 2021). This precision is particularly advantageous in the complex and delicate environment of the oral cavity, where preserving tooth structure and minimizing collateral damage is critical. Furthermore, PTT’s ability to generate heat at specific depths reduces the production of exopolysaccharides—the main component endowing biofilm architecture and stability (Yao W. et al., 2025). This mechanism aids in the disintegration of biofilms and ensures effective penetration, addressing a major limitation of traditional antimicrobial therapies. Emerging studies have also highlighted the potential of PTT to synergize with other therapeutic modalities (Xu X. et al., 2022; Li et al., 2024c). One key feature of PTT, its efficient thermal generation at desired locations, can enhance combination therapies in various ways. For instance, PTT can be combined with photodynamic therapy (PDT) or antimicrobial agents, where the photothermal effect promotes the controlled release of these agents (Xu H. et al., 2022; Pan et al., 2025) or enables on-demand modality switching between PTT and PDT (Zhang Y. et al., 2024), thereby boosting their efficacy in eradicating biofilms. An adaptive supramolecular nanoformulation (ZnPcS4@GC5AF5, GFZ) switchable from PTT to PDT under the trigger of adenosine triphosphate (ATP) was reported. The activation of the photothermal properties of GFZ through visible irradiation led to bacterial cell membrane rupture and intracellular ATP release. Subsequently, ATP reduced the photothermal activity (low state) and restored the photodynamic activity (ON state). A large number of reactive oxygen species (ROS)were generated while avoiding high local temperatures, which not only resulted in eradicating pathogenic bacteria biofilms but also minimized heat damage to normal pulp tissues (Figure 3B) (Zhang Y. et al., 2024).

Despite these promising advances, several challenges remain in translating PTT into clinical practice for caries treatment. Key issues include optimizing the parameters of light irradiation (e.g., wavelength, intensity, and duration) to achieve effective biofilm eradication without causing thermal damage to oral tissues. Additionally, PTAs’ long-term safety and biocompatibility must be rigorously evaluated to confirm their suitability in the oral cavity. The prevention and management of dental caries represent a protracted process, so further research is also needed to investigate the potential of PTT in preventing caries’ recurrence and addressing the complex microbial ecology of dental biofilms. Developing composite materials capable of inhibiting the proliferation of cariogenic bacteria and facilitating the remineralization of early-stage demineralized dental tissues constitutes a promising research trajectory for the future (Zhu et al., 2022). In conclusion, PTT represents a groundbreaking approach to combating dental caries, offering a combination of precision, efficacy, and minimal invasiveness that addresses the limitations of current therapies. As research in this field continues to advance, PTT holds the potential to revolutionize the prevention and treatment of dental caries, ultimately improving oral health outcomes and alleviating the global burden of this pervasive disease.

## 3 PTT for endodontics

The dental pulp comprises sterile connective tissue and is protected by the surrounding enamel, dentin, and cementum (Pohl et al., 2024). Exposure resulting from factors including trauma, dental caries, or tooth wear can precipitate endodontics, characterized by symptoms such as pain, sinus tracts, and swelling (Karamifar et al., 2020). The elimination of bacteria and their biofilms assumes a pivotal role in the treatment of endodontics (Neelakantan et al., 2017). In clinical practices, root canal therapy (RCT) represents a commonly employed approach for removing microorganisms that instigate or exacerbate this ailment (Burns et al., 2022; Huang et al., 2024). Although biomechanical root canal preparation and chemical sterilization of irrigants could effectively eradicate the microbes, achieving thorough debridement and eradicating tenacious infections persist as formidable challenges in root canal treatment (Moradi Eslami et al., 2019). Additionally, High concentrations of chemical irrigants may cause serious damage by irritating periodontal soft and periapical tissues (Xu H. et al., 2022). *Enterococcus faecalis* (*E. faecalis*) is a key bacterial species frequently isolated from root canals afflicted with refractory endodontic infections, contributing to 20%–70% of RCT failures (Manoil et al., 2023). This is attributed to its capacity to form biofilms which can adapt to external alterations as an integrated entity (Pourhajibagher et al., 2018; Cao et al., 2021). Consequently, various studies aim to explore novel materials, encompassing irrigants and intracanal dressings, to eliminate *E. faecalis* in the biofilm phase (Aksoy et al., 2020; Cao et al., 2021; Duan et al., 2022; Galdámez-Falla et al., 2022; Feng et al., 2024). During PTT, hyperthermia aids in biofilm disintegration and induces bacterial demise (Mei et al., 2023; Zhou et al., 2024). Crucially, it remains confined within the root canal, as tooth hard tissues impede the complete transfer of heat to the periodontal tissues, thereby reducing potential damage to these tissues (Duan et al., 2022). Studies have demonstrated that PTT exhibits remarkable efficacy against *E. faecalis* and its biofilms without compromising dentin strength, supporting its potential as a prospective antibacterial therapy during RCT (Castillo-Martínez et al., 2015; Khantamat et al., 2015; Bermúdez-Jiménez et al., 2020; Galdámez-Falla et al., 2022).

**FIGURE 4:**
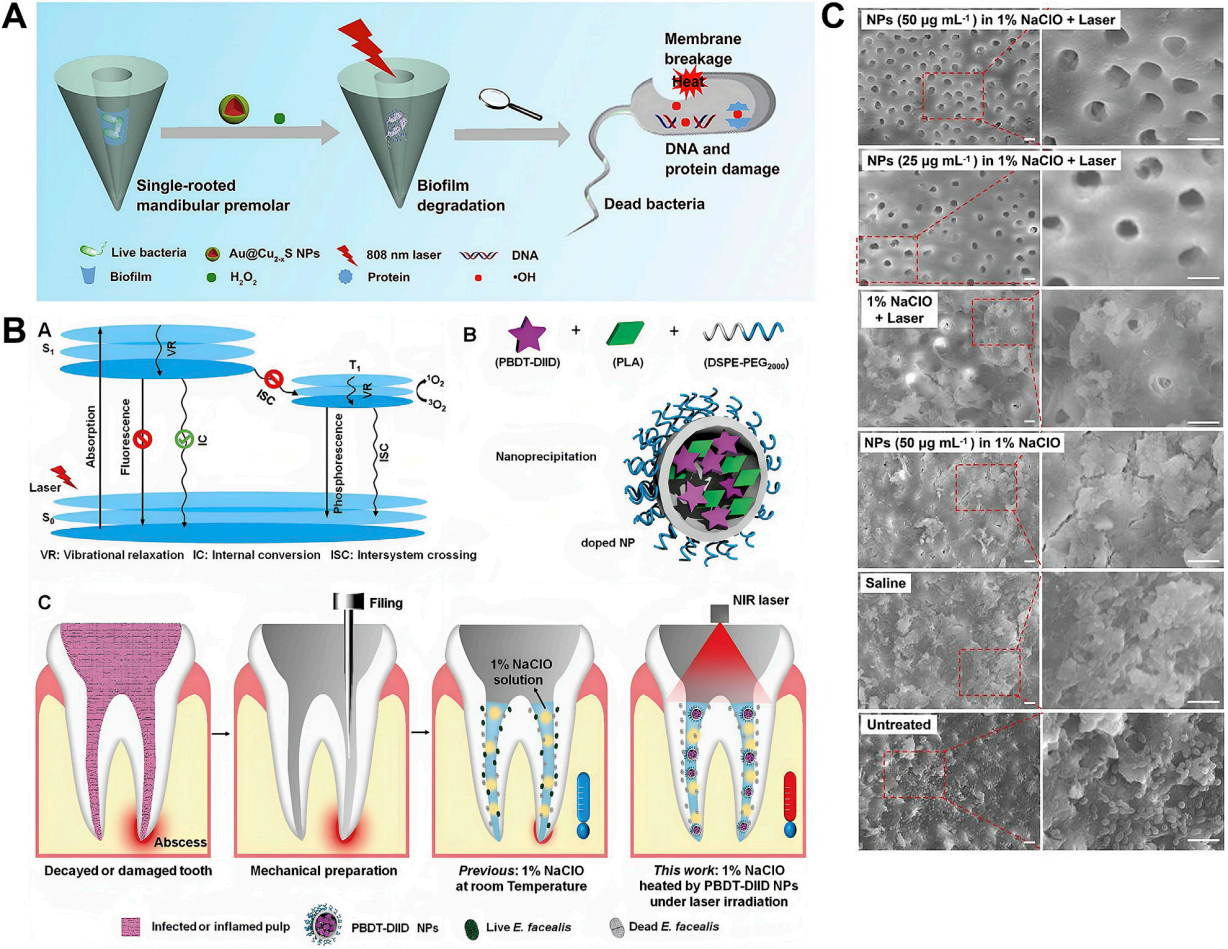
Photothermal therapy (PTT) for endodontics. **(A)** Schematic illustration of Au@Cu2-xS nanoparticles with NIR photothermal and peroxidase (POD) catalytic activities for antibiofilm-oriented root canal therapy. Reproduced with permission (Cao et al., 2021). Copyright 2021, Elsevier Group. **(B)** Schematic diagram illustrating the heating of a 1% NaClO solution under 808 nm laser irradiation in the presence of PBDT-DIID nanoparticles to enhance intracanal sterilization. **(C)** Representative SEM images of the middle of the sample tooth in different groups. Reproduced with permission (Duan et al., 2022). Copyright 2022, John Wiley and Sons Group.

The thermal generation of PTT not only directly inhibits *E. faecalis* and its biofilms, but can also be combined with root canal conventional irrigants, such as sodium hypochlorite (NaClO) and hydrogen peroxide (H_2_O_2_), to augment the overall efficacy of root canal disinfection. Cao et al. (2021) constructed Au@Cu_2-x_S NPs by integrating Cu_2-x_S with peroxidase-like activity and Au NPs with photothermal effect to augment the capacity to eliminate biofilms. It not only exhibits strong photothermal activity but also catalyzes H_2_O_2_ to generate hydroxyl radicals (·OH), which are more effective for biofilm degradation. Mechanistic studies demonstrated that the treatment effectively degrades proteins and polysaccharides—the primary components of biofilm EPS. The synergistic strategy combining PTT and peroxidase-like catalytic treatment with H_2_O_2_ holds significant potential for eradicating bacteria and biofilms within root canals (Figure 4A). Heating 1% NaClO—another irrigant extensively used clinically—within the root canal during PTT also significantly enhances its antibacterial efficacy (Abou-Rass and Oglesby, 1981; Tosić et al., 2016). A temperature increase of <10 °C on the external root surface achieved 99.7% antimicrobial efficacy against *E. faecalis* using heated 1% NaClO solution (Figure 4B) (Duan et al., 2022). Additionally, scanning electron microscopy (SEM) reveals that the teeth treated in the experimental group exhibit regular exposure of dentinal tubules. Conversely, the dentin in the control groups exhibited a rough surface, characterized by a profusion of bacteria and smear layers, with the majority of dentinal tubules remaining occluded (Figure 4C) (Duan et al., 2022). These studies manifested the potential of safely and efficaciously improve the RCT outcome by heating the irrigants.

Current research on PTT for endodontic diseases focuses primarily on its antibacterial role as an adjunct to root canal therapy, predominantly targeting *E. faecalis*. As effective treatments for refractory and recurrent root canal infections remain lacking, PTT offers a promising therapeutic approach. Nevertheless, the tooth root canal is complex and contains many small branching canals. The root canal biofilm is a very complex, organized entity (Neelakantan et al., 2017). Single-rooted mandibular premolar models and monospecies biofilms used in previous studies may oversimplify the root canals and the ecological phenomenon of biofilms. They may not truly reflect the results achievable in the clinical scenario. After the photothermal material is injected into the root canal and exerts its function, the challenge of effectively removing it without impeding subsequent root canal filling represents major hurdle confronting research in this field.

## 4 PTT for periodontitis

Among all oral infectious diseases, the PTT for periodontitis has garnered the most extensive attention. Periodontitis represents a chronic inflammatory disorder instigated by bacteria (Kwon et al., 2021). The establishment of pathogenic bacteria within subgingival dental plaque provokes the host immune response, resulting in the generation of a significant amount of ROS and subsequent oxidative stress (Sczepanik et al., 2020; Kwon et al., 2021; Iniesta et al., 2023). Consequently, this process leads to the degradation of tooth-supporting tissues, eventually resulting in the development of periodontal pockets, alveolar bone resorption, and subsequent tooth loosening (Kuboniwa et al., 2017; Tóthová and Celec, 2017; Heitz-Mayfield, 2024). Currently, in clinical practice, mechanical debridement and antibiotics are commonly employed (Mombelli, 2018; Cobb and Sottosanti, 2021). Nevertheless, in most cases, mechanical debridement proves arduous to comprehensively eliminate periodontitis infections within deep-seated periodontal pockets, furcation, and irregular root surface regions (Umeda et al., 2004). Additionally, the protracted administration of antibiotics engenders numerous issues, such as the development of drug-resistant bacteria, bacillary dysentery, and gastrointestinal disorders (Rams et al., 2020). Beyond bacterial factors, biofilm-induced immune dysregulation constitutes another major contributor to impaired bacterial clearance and disease persistence in periodontitis. Consequently, periodontitis treatment represents a key research focus in dentistry, with current strategies targeting not only antibacterial action but also anti-inflammatory effects and periodontal regeneration (Kong et al., 2023; Wang F. et al., 2023; Li T. et al., 2024; Li Z. et al., 2024; Yang et al., 2025). PTT offers distinct advantages for periodontitis treatment, as it not only enables efficient and safe bacterial elimination but also promotes cell proliferation, angiogenesis, wound healing, and bone regeneration—key factors in periodontal recovery (Zhang et al., 2021). More significantly, it can be integrated with PDT, chemical dynamic therapy (CDT), antibacterial agents, and bioactive materials to construct a material system featuring multi-functional synergy in antibacterial, anti-inflammatory, and tissue-regeneration functions (Shi et al., 2021; Bai et al., 2022; Wang H. et al., 2023; Lin et al., 2024; Wu et al., 2025).

Unlike dental caries or endodontic treatments—which involve heat-tolerant hard tissues—periodontitis affects the thermally sensitive periodontium. Combination therapy—a well-established paradigm in antimicrobial treatment—enhances therapeutic efficacy by integrating distinct therapeutic mechanisms beyond the capabilities of individual monotherapies (Wang N. et al., 2022). This approach enables superior outcomes at reduced thermal dosages. Mild hyperthermia (<45 °C) enhances the bactericidal efficacy of antibiotics by inhibiting enzyme activity, while preserving surrounding tissue integrity. tissues (Gao et al., 2019). The synergy between PTT and antibiotics such as tetracycline (TC) (Zhang et al., 2020) and minocycline (Lin et al., 2020; Wang X. et al., 2023) represents a strategic approach for an efficacious periodontal antibacterial therapy. To enhance drug delivery efficiency and curtail systemic harm, drug delivery systems (DDS) are frequently utilized to administer antibiotics (Lin et al., 2020; Zhang et al., 2020; Shi et al., 2021). Hydrogels, widely employed in DDS, can conform to the irregular morphology of periodontal pockets and enhance the retention rate of the released drugs at the local infection site. The heat of PTT can stimulate and trigger the controlled release of drugs within the DDS, thereby achieving synergistic sterilization through the combined action of antibiotics and photothermal effects (Lin et al., 2020; Zhang et al., 2020). Beyond antibiotics, PTT could synergize with alternative antibacterial strategies—including Ag^+^ (Wang F. et al., 2023),. PDT (Bai et al., 2022; Gao et al., 2023; Wu et al., 2023), CDT (Chen Q. et al., 2023; Dai et al., 2023; Lin et al., 2024), and gas therapy (Dai et al., 2023; Li Z. et al., 2024)—to enhance periodontal biofilm eradication. The synergistic integration of PDT and PTT, activated by a single 808 nm NIR source, amplifies antibacterial efficacy while reducing both drug dosage and laser energy requirements (Qi et al., 2022). This dual-modal approach enhances bacterial elimination beyond monotherapies: PTT-induced hyperthermia disrupts membrane integrity, facilitating deeper penetration of ROS generated through PDT to inflict lethal oxidative damage (Wang R. et al., 2022; Wu et al., 2023). Highly toxic ·OH generated by CDT exhibits potent destructive effects on bacterial biofilms and cell membranes, demonstrating significant efficacy against bacterial infections (Guo et al., 2020). Notably, these ·OH radicals critically deplete ATP levels, inhibiting heat shock proteins and reducing bacterial heat resistance (Chen et al., 2020; Wang F. et al., 2023). This thereby enhances the efficiency of PTT, highlighting a promising single-material solution for concurrent CDT and PTT. Gas therapy represents a novel, promising strategy for targeting deep infections in periodontal tissues. Nitric oxide (NO) has demonstrated outstanding antimicrobial efficacy and the ability to combat resistance linked to bacterial biofilms (Li Z. et al., 2024). It could increase the sensitivity of the bacteria to heat and promote tissue healing by stimulating angiogenesis and alleviating the damage caused by periodontitis (Yuan et al., 2020; Dai et al., 2023). When combined with PTT, this approach demonstrates significant synergistic efficacy in the treatment of periodontitis (Dai et al., 2023; Li T. et al., 2024).

**FIGURE 5:**
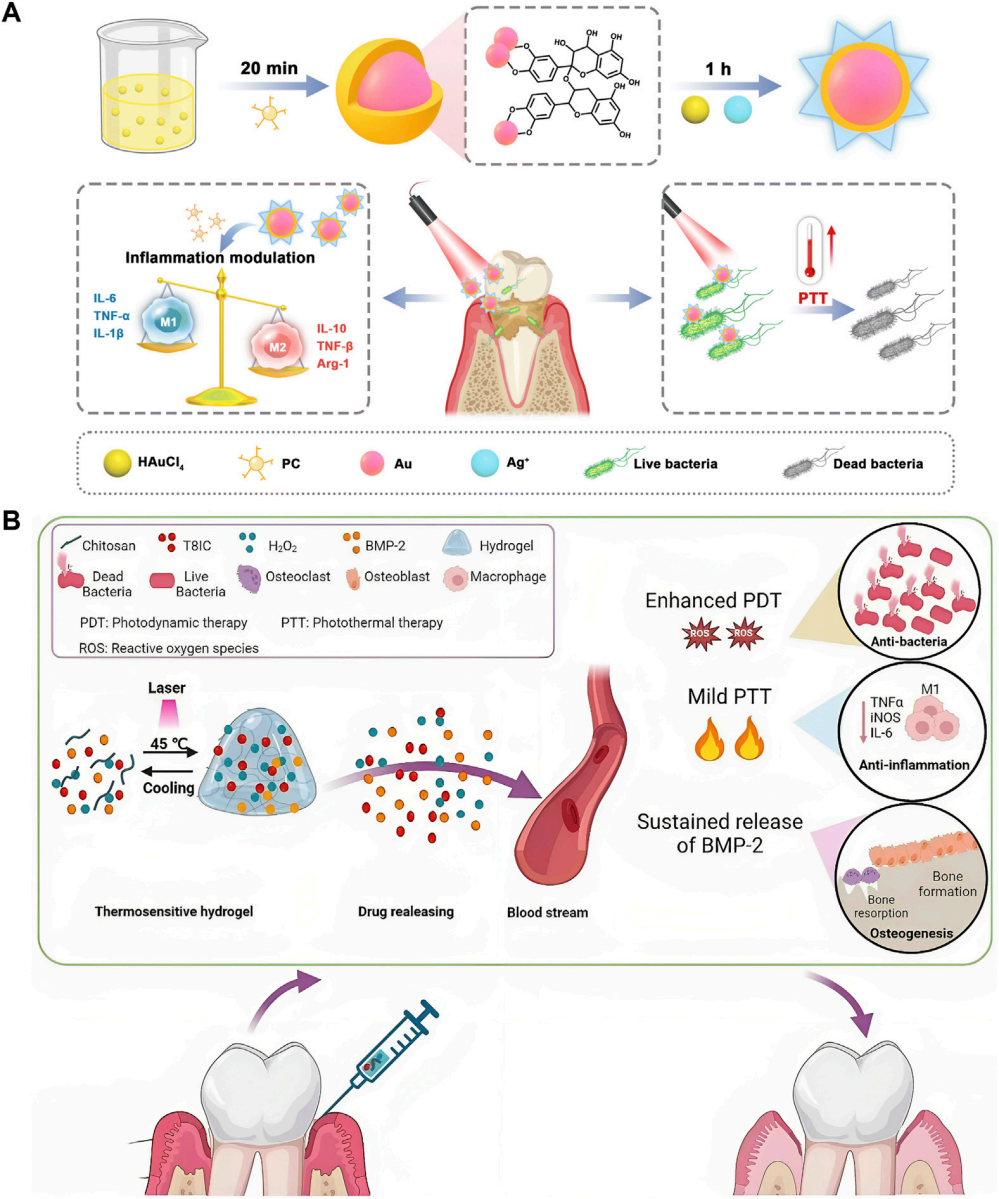
Photothermal therapy (PTT) for periodontitis. **(A)** Schematic illustration depicting the synthesis principle and therapeutic mechanism of AuAg-PC nanoparticles in treating periodontitis. Reproduced with permission (Wang F. et al., 2023). Copyright 2023, Royal Society of Chemistry. **(B)** Schematic illustration of thermosensitive and injectable hydrogel with T8IC, H_2_O_2_, and bone morphogenetic protein-2 (BMP-2). Reproduced under Creative Commons CC BY license (Wang P. et al., 2023). Copyright 2023, The Author(s), Published by Springer Nature Group.

Bacterial infection might be the primary cause of inflammation’s initial stages, but the host’s immune inflammatory response is responsible for promoting periodontitis (Hajishengallis, 2014). To treat periodontitis thoroughly, regulating host immunity is also crucial in addition to clearing the biofilm in the disease area (Qi et al., 2022; Wang N. et al., 2022; Chen Q. et al., 2023). Proanthocyanidins (PCs), a class of natural phenolic compounds, demonstrate efficacy in impeding the elevation of ROS inhibiting inflammatory factors, and regulating macrophage polarisation in periodontal disease sites (Gil-Cardoso et al., 2019; Kim et al., 2019; Wang H. et al., 2022; Zhang et al., 2023). A nanocomposite named AuAg-PC NPs was synthesized with PCs as a reducing agent. Biofilms can be eradicated through Ag^+^-synergistic PTT, whereas PCs demonstrate the capacity to eliminate ROS and modulate tissue self-healing via the PI3K/Akt signaling pathway. Hence, the nanocomposites can eradicate periodontal pathogens and restore the immune regulation environment (Figure 5A) (Wang F. et al., 2023). Additionally, baicalein (BA) (Tian et al., 2022), nitric oxide (NO) (Qi et al., 2022), PB nanozymes (Wang P. et al., 2023; Li Z. et al., 2024), ceria (CeO_2_) (Li T. et al., 2024), rapamycin Xiao et al., 2024) and dimethyl fumarate (DMF) (Li T. et al., 2024) have been employed to modulate the detrimental innate inflammatory responses triggered during persistent infections. Considering that the destruction of periodontal soft tissues and the resorption of alveolar bone induced by periodontitis are irreversible processes, periodontal tissue regeneration is crucial for treating periodontitis (Yao H. et al., 2025). Although PTT can promote osteogenesis, monotherapies are often insufficient to elicit an adequate therapeutic response—and PTT is no exception. Recently, tissue engineering has proffered new prospects for repairing periodontal tissue defects in patients with periodontitis (Hussain et al., 2022; Wang P. et al., 2023). A thermosensitive and injectable hydrogel with a three-dimensional (3D) network architecture was employed as a delivery system for the controlled release of osteoinductive agents (BMP-2) and phototherapy agents (T8IC and H_2_O_2_). PTT combined with PDT exhibited excellent bactericidal effects while sustained release of BMP-2 and mild temperature (45 °C) induced osteogenesis (Figure 5B) (Wang P. et al., 2023). An appropriate concentration of Cu^2+^ promotes the proliferation and osteogenic differentiation of bone marrow mesenchymal stem cells (BMSCs) (Burghardt et al., 2015). Nanomaterials such as copper sulfide (CuS) nanoparticles leverage this biological activity while exhibiting strong NIR absorption and exceptional PCE, enabling their use as potent PTAs (Yang et al., 2025). This dual functionality facilitates simultaneous spatiotemporal antibacterial action and alveolar bone regeneration.

Notable progress has been achieved in the research on PTT for periodontitis. The research spans three key aspects: antibacterial, anti-inflammatory, and tissue-regeneration. Each function can synergize with the others, yielding favorable outcomes. However, we have not yet seen a multifunctional material or system integrating all of them, and the development of triple-functional materials or systems represents a future research trajectory. Although current photothermal conversion materials, such as gold nanorods, exhibit excellent biocompatibility, their long-term retention in the body may hinder periodontal tissue regeneration and affect overall health. Future research should focus on developing photothermal materials that can be metabolized and cleared by the body to avoid potential adverse effects. Additionally, previous studies predominantly utilize near-infrared region I (NIR-I, 650–1000 nm) lasers; near-infrared region II (NIR-II, 1000–1700 nm) which offers deeper tissue penetration into the periodontal pocket and improved precision in targeting periodontal lesions is a promising direction for future research (Luo et al., 2025).

## 5 PTT for peri-implantitis

Peri-implantitis constitutes a pathological condition associated with dental plaque that occurs in the tissues surrounding dental implants (Giok et al., 2024). The hallmark of this condition includes inflammation of the peri-implant mucosa and concomitant supporting bone loss (Berglundh et al., 2018; Schwarz et al., 2018). Inflammatory manifestations, bleeding or hyperemia upon probing, an augmentation in probing depth, and radiographic indications of bone resorption constitute the typical clinical features of peri-implantitis (Diaz et al., 2022). Unlike natural teeth, implants lack a periodontal ligament to separate the inflammatory cell infiltrate from the crestal bone (Carcuac et al., 2013). Therefore, peri-implantitis progresses faster than periodontitis around teeth. In the absence of effective intervention, the inflammatory process progressively damages osseointegration and ultimately causing implant mobility and loss (Daubert et al., 2015; Derks et al., 2016). Furthermore, the peri-implant microbiome and biofilm composition differ from those around natural teeth, making peri-implantitis management more challenging and less predictable than periodontitis treatment (Koyanagi et al., 2013; Wu L. et al., 2019). Clinically, the management of peri-implantitis bears resemblance to that of periodontitis, predominantly relying on the mechanical elimination of biofilms and the administration of antibiotics (Giok et al., 2024). However, due to the inaccessibility of infected implant surfaces and the potential for mechanical debridement to damage implant topography, effective biofilm eradication and re-osseointegration remain clinically challenging (Wang et al., 2020; Munakata et al., 2022; Ichioka et al., 2023). Therefore, preventing peri-implantitis is clinically paramount—significantly more critical than treatment.

**FIGURE 6:**
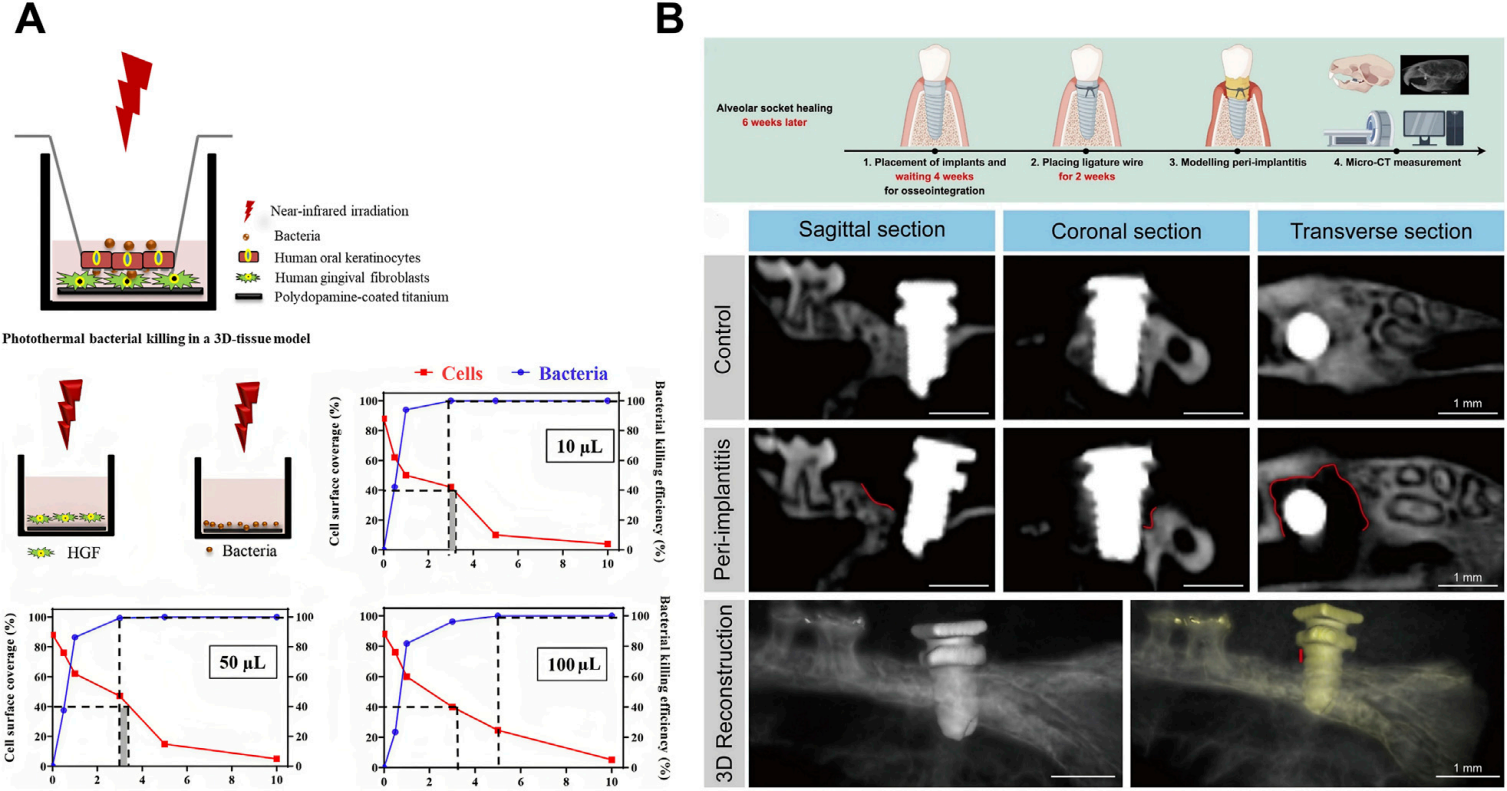
Photothermal therapy (PTT) for peri-implantitis. **(A)** Schematic illustration demonstrating photothermal bacterial killing in a 3D tissue model and surface coverage of human gingival fibroblasts (HGFs) and the eradication of *Staphylococcus aureus* (*S. aureus*) upon NIR irradiation of PDA nanoparticle coated titanium surfaces in monocultures. Samples were immersed in varying volumes of DMEM-HG medium (for HGFs) and PBS (for staphylococci), as illustrated in the schematics. The dotted lines demarcate NIR irradiation times considered acceptable for preserving tissue integration (>40% cell surface coverage; red data) and ensuring significant bacterial killing (>99.9%; blue data). The gray shading indicates the range of acceptable irradiation times that satisfy both criteria. Reproduced under Creative Commons CC BY-NC-ND 4.0 license (Ren et al., 2020). Copyright 2020, American Chemical Society. **(B)** Schematic diagram, Micro-CT, Hematoxylin-Eosin (HE) staining, and tartrate-resistant acid phosphatase (TRAP) staining of *in vivo* modeling of peri-implantitis in mice. Reproduced with permission (Liu et al., 2025). Copyright 2025, Elsevier Group.

The development of peri-implantitis begins with planktonic bacterial adhesion to implant surfaces (Osman et al., 2022). While titanium alloys and zirconia are common dental implant materials, neither exhibits inherent antibacterial activity (Pieralli et al., 2017; W. Nicholson, 2020; Chen et al., 2021). Consequently, enhancing the antimicrobial functionality of implants is critical to mitigate peri-implantitis. Surface modifications can profoundly alter the micro/nanotopography and chemical composition of titanium implants, enhancing hydrophilicity, mechanical stability, osseointegration capacity, and antibacterial efficacy (Sun et al., 2023; Gkioka and Rausch-Fan, 2024; Yu Y. M. et al., 2024). When irradiated with NIR light, dental implants coated with graphene oxide (GO) (Park et al., 2023) or PDA nanoparticles (Ren et al., 2020) demonstrate reduced adhesion of *S. mutans* and *Porphyromonas gingivalis* (*P. gingivalis*). Despite their antibacterial efficacy, photothermal coatings risk collateral tissue damage through heat dissipation near infection sites, potentially compromising healthy peri-implant tissue integration (Werner et al., 2009). Ren et al. (2020) devised a model wherein keratinocytes were cultured on a membrane filter within a transwell system while fibroblasts adhered to a titanium surface beneath the membrane. This model could be used to investigate the previously uninvestigated risk of collateral tissue damage from photothermal coatings on implant surfaces (Figure 6A). The use of novel biomaterials represents another strategy. Similar to periodontal therapy, this approach targets bacterial elimination and reduces inflammatory responses through immunoregulation (Xue et al., 2023; Liu et al., 2025). A critical distinction, however, is the requirement for a firm biological seal between the abutment and the gingival epithelium (Mahmoud et al., 2019). This seal is essential to prevent bacterial invasion and subsequent marginal bone loss (Fischer et al., 2022). Additionally, dental implants must achieve osseointegration with alveolar bone post-implantation (Chen et al., 2024). Xue et al. (2023) proposed a multipurpose photothermal strategy that uses Si/P/F-doped TiO_2_ to address these challenges through dual functionality: exhibiting strong photothermal response and NIR-triggered F^−^ release. The resulting hyperthermia-F^-^ synergy disrupts *Staphylococcus aureus* (*S. aureus*) by reducing ATP synthesis, increasing membrane permeability, and generating ROS that oxidize cellular components to cause bacterial death. Concurrently, mild hyperthermia with released ions enhances gingival epithelial hemidesmosome formation and osteoblast activity. Another critical distinction in the field of peri-implantitis is the complexity of establishing animal models. Several *in vivo* studies of dental peri-implantitis have employed mouse femoral peri-implantitis models (Xiao et al., 2024; Wang et al., 2025); however, these models fail to accurately replicate the clinical condition of dental peri-implantitis within the alveolar bone (Zhang J. et al., 2024). The “ligature model” in alveolar bone mimics naturally occurring peri-implantitis and is suitable for studying the disease (Carcuac et al., 2013). The optimal timing for implant placement in mouse alveolar bone to establish a murine peri-implantitis model remains a contentious issue due to the limited understanding of the anatomical structure and physiological state of the alveolar bone after implant placement (Tzach-Nahman et al., 2017; Wong et al., 2018). Micro-CT and histological sectioning techniques suggested 6 weeks after the extraction of the maxillary first molar might be the appropriate time for implant placement (Figure 6B) (Liu et al., 2025). This finding offers significant data supporting the development of the murine peri-implantitis model.

Given the escalating prevalence of dental implants in dental prosthodontics clinical practice, it is anticipated that peri-implantitis will garner increasing attention. As a non-invasive and non-antibiotic-resistant antibacterial strategy, PTT holds great promise in preventing and treating peri-implantitis. Notably, when integrated with bone regeneration strategies, it can substantially promote the osseointegration process while preventing postoperative infection and enhancing the success rate of implant surgery. The current research bottleneck lies in determining how to minimize or eliminate collateral photothermal damage to healthy tissue cells in the peri-implant region while effectively eradicating bacteria through photothermal action. With the establishment of suitable animal models, future research in this field will accelerate, leading to significant advances.

## 6 PTT for other oral infectious diseases

**FIGURE 7:**
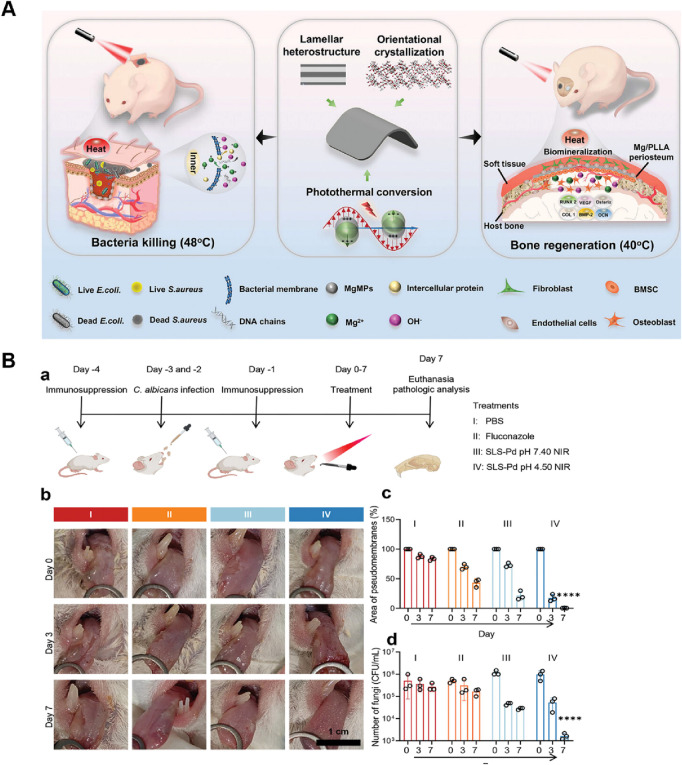
PTT for infectious bone defect and *candida* infection. **(A)** Schematic illustration of the self-reinforced Mg/PLLA composite membrane with lamellar heterostructure working as a periosteum for on-demand bacteria inhibition and rapid bone reconstruction. Reproduced with permission (Wang F. et al., 2023). Copyright 2022, John Wiley and Sons Group. **(B)** MPN-Pd-mediated system for the treatment of oral candidiasis. (a) Workflow of the *in vivo* experiment. (b) Digital images of oral candidiasis models under different treatments. (c) Quantitative analysis of the pseudomembrane area. (d) Viability of *Candida albicans* (*C. albicans*) evaluation. Reproduced with permission (Chen et al., 2023a). Copyright 2023, John Wiley and Sons Group.

Infectious bone defects (IBD) collectively refer to a class of diseases characterized by tenacious infection, persistent inflammation, bone destruction, impaired blood supply, and a protracted course of diseases, making them particularly challenging to manage (Han et al., 2024). It can be caused by jaw osteomyelitis, trauma, postoperative infection of tumors, etc (Dong et al., 2017). Clinical treatment strategies typically encompass antibiotic administration, excision of necrotic bone fragments, debridement procedures, and transplantation of bone grafts (Qian et al., 2023; Han et al., 2024). Nevertheless, antimicrobial overuse drives the evolution of drug-resistant strains (Hu et al., 2024). Moreover, requiring bone graft implantation post-infection eradication significantly prolongs treatment duration. Therefore, developing biomaterials that simultaneously deliver antibacterial functionality and personalized osteogenic capabilities is imperative. The photothermal effect delivers dual benefits: conferring antibacterial activity while using moderate local heating to upregulate key genes (e.g., osteogenesis-related genes) that promote tissue regeneration (Avci et al., 2013; Ma et al., 2020). Wang W. et al. (2023) developed a lamellar heterostructured Mg/PLLA composite periosteum membrane via an accumulative rolling method for application at bone defect sites. A consistent supply of Mg^2+^ activates key extracellular matrix proteins and transcription factors implicated in bone regeneration and angiogenesis. The photothermal effect of Mg microparticles can eliminate bacteria while further enhancing bone marrow-derived mesenchymal stromal cells (BMSCs) differentiation. Although overheating risks inducing apoptosis in both bacteria and healthy cells, longitudinal analysis revealed converging cell densities between composite membrane treated and control groups over time. This demonstrates that strategically controlled PTT ultimately favors tissue repair over thermal damage. Consequently, the PTT-enhanced composite periosteum achieved on-demand antibacterial efficacy and exceptional endogenous vascularized bone regeneration (Figure 7A). Beyond artificial periosteum, research has extended to 3D-printed hydrogels for tissue regeneration (Nie et al., 2022). These studies indicate that the application of PTT in the field of biomedical engineering holds great promise.

Beyond its efficacy against oral bacterial infections, PTT has demonstrated effectiveness against other microbial infections, including fungal infections. *Candida albicans* (*C. albicans*) is a primary etiological agent for most nosocomial infections affecting immunocompromised patients, and emerging multidrug resistance has made it an urgent threat (Arendrup and Patterson, 2017). Oropharyngeal candidiasis represents a form of oral candidal infection with a higher prevalence in individuals with conditions such as diabetes mellitus, immunodeficiency, and xerostomia (Stoopler et al., 2024). Similarly, it is associated with *C. albicans* biofilms on the oral mucosa. The intrinsic resistance of biofilms to antifungal agents has augmented the challenges associated with effective antifungal treatment. Chen et al. (2023a) developed a metal-phenolic network with Pd nanoparticle nodes (MPN-Pd) and found that *C. albicans* is more sensitive to hyperthermia than bacteria like *E. faecalis* and *S. mutans* which might be attributed to the fungal membrane containing dipalmitoylphosphatidylcholine phospholipid molecules that are more sensitive to temperature. The histological evaluation of mouse oral *candida* infection model indicates that the PTT is effective in the therapeutic goal of treating oropharyngeal candidiasis by eradicating *C. albicans* in the oral cavity, while showing no sign of collateral damage (Figure 7B). However, the current research on the antifungal application of PTT remains in its nascent stages, with limited experimental and clinical data currently available. Viral infections cause oral infectious like herpetic stomatitis. Theoretically, PTT also has the potential to be used for antiviral therapy, as viral structures and proteins are also prone to denaturation and inactivation at high temperatures (Bai et al., 2023; Li B. et al., 2024). Given PTT’s remarkable antibacterial prowess and compatibility with other treatment modalities or bioactive materials, it is expected to be used to treat a broader spectrum of oral infections.

## 7 Summary and outlook

PTT heralds a paradigm shift in the prophylaxis and therapeutic strategies for oral infections. It proffers an efficacious, precise, and minimally invasive alternative to antibiotics. By capitalizing on the potency of light and heat, PTT surmounts the limitations inherent in extant therapies, such as the burgeoning issue of antibiotic resistance and the propensity for tissue damage. Simultaneously, it furnishes a platform conducive to innovative and multifarious applications. As research within this domain continues to burgeon, PTT offers significant potential to revolutionize the management of oral infections charting a course towards more efficacious and sustainable solutions in oral healthcare. Critically, PTT demonstrates not only potent antibacterial efficacy but also significant potential for promoting tissue regeneration. Its compatibility with other therapeutic modalities enables synergistic treatment outcomes—particularly valuable for managing periodontitis and infectious bone defects where restoring biological function extends beyond mere antibacterial control.

Despite its considerable potential, PTT’s clinical translation markedly lags behind that of its counterpart, PDT, with scarce clinical trials, and a range of challenges must be surmounted to fully actualize its clinical implementation for treating oral infections. The dual objectives of potent bactericidal effects and minimal collateral tissue damage pose an inherent trade-off, which may be addressed by improving targeting specificity. This underscores the need for advanced intelligent drug delivery systems and highly precise laser irradiation with deep-tissue penetration capability. Furthermore, combining PTT with adjuvant therapies to enhance bacterial photosensitization offers an alternative viable approach. Moreover, long-term biocompatibility and safety of PTAs necessitate comprehensive assessment to attenuate potential risks, such as tissue inflammation or systemic toxicity. The clinical translation of PTAs hinges on their long-term biosafety and effective clearance to mitigate the toxicity risk from bioaccumulation. To address this, key strategies focus on either biodegradability or renal clearance. For metallic PTAs like gold, which are poorly biodegradable, engineering ultrasmall, renally-clearable (<5 nm) nanoparticles offers a promising solution (Hwang et al., 2014; Tang et al., 2014). Alternatively, designing for biodegradability is a major focus. This includes inherently biodegradable inorganic materials like black phosphorus, which degrades into harmless phosphates, and carbon-based materials (e.g., GO) that can be broken down by enzymes (Lalwani et al., 2014). Organic materials often show superior biocompatibility; the FDA-approved dye indocyanine green (ICG) provides a clinical benchmark with its rapid hepatobiliary clearance, while engineered polymers (semiconducting polymer nanoparticles, SPNPs) can be designed with cleavable bonds (Lyu et al., 2018; Della Pelle et al., 2021). Ultimately, this focus on creating intentionally degradable or clearable nanoparticles is the critical step toward bringing PTT from preclinical studies to clinical reality. Finally, a significant barrier to the clinical translation of PTT is the lack of standardized parameters across preclinical studies. This challenge is formidable, extending beyond just light exposure conditions. A review of the literature reveals considerable variability in irradiation, with typical parameters involving an 808 nm laser at a power density of 0.5-3 Wcm^-2^ for 5–10 min, aiming for temperatures of 55 °C–60 °C for conventional PTT or a milder ∼41 °C–43 °C for mild PTT. Furthermore, given that this research is still largely in the preclinical stage, different laboratories employ unique nanoparticle platforms and diverse infection models. This heterogeneity makes it exceedingly difficult to directly compare the therapeutic efficacy of different photothermal systems. Therefore, establishing standardized protocols that encompass not only irradiation parameters but also the class of nanomaterial and the type of infection being modeled is imperative to accelerate the clinical translation of this promising therapeutic modality.

In light of this, future research endeavors regarding the application of PTT in oral infections, encompassing dental caries, endodontics, periodontitis, and peri-implantitis, should center on the following aspects: ⅰ. Establish experimental models that can duplicate the complexity of biofilms to evaluate the antibacterial efficacy of PTT comprehensively. The mechanism of PTT against dental plaque biofilms also needs to be further studied. ⅱ. Develop photothermal materials that are smart-responsive, degradable, or can be cleared by body metabolism to improve the biosafety of PTT. ⅲ. Probe into applying NIR-II lasers in deep-seated oral tissues to augment the precision of treatment. ⅳ. Fortify interdisciplinary integration, promote the combinatorial utilization of PTT with traditional antibacterial, immunomodulatory, and tissue-regeneration strategies, and engineer multifunctional materials. ⅴ. Facilitate large-scale clinical trials, standardize treatment parameters, evaluate long-term biosafety, and ultimately propel its clinical translation. Future research should also be directed towards elucidating the interplay between PTT and the oral microbiota, especially its implications for non-pathogenic commensal bacteria. Preserving the eco-logical equilibrium of the oral microbiota is pivotal for upholding overall oral health and forestalling diseases associated with dysbiosis. Additionally, developing cost-effective and scalable PTT systems is imperative for its widespread clinical deployment, particularly in resource-constrained settings. In summary, PTT presents a highly promising approach to the treatment of oral infections, replete with substantial potential for clinical translational applications. With the evolution of multi-disciplinary convergence, it may emerge as a novel approach for combating oral-related infections, thereby conferring greater benefits to humanity.

## Glossary

3D: Three-dimensional
Alg: Sodium alginate
ATP: Adenosine triphosphate
ATRP: Atom transfer radical polymerization
AuNPs: Gold nanoparticles
AuNRs: Gold nanorods
BA: Baicalein
BMP-2: Bone morphogenetic protein-2
BMSCs: Bone marrow-derived mesenchymal stromal cells
BP: Black phosphorus
*C. albicans*: *Candida albicans*
CDT: Chemical dynamic therapy
DDS: Drug delivery systems
*E. coli*: *Escherichia coli*
*E. faecalis*: *Enterococcus faecalis*
eNOS: Endothelial nitric oxide synthase
EPL: Epsilon-polylysine
EPS: Extracellular polymeric substances
*F. nucleatum*: *Fusobacterium nucleatum*
GelMA: Gelatin methacrylate
GNC: Gold nanocages
GO: Graphene oxide
HE: Hematoxylin-Eosin
HGFs: Human gingival fibroblasts
HSP90: Heat shock protein 90
IBD: Infectious bone defects
ICG: Indocyanine green
MgMps: Mg microparticles
MPB: Mesoporous Prussian blue
MPN: Metal-phenolic networks
mPTT: Mild photothermal therapy
MRSA: Methicillin-resistant *Staphylococcus aureus*
MSC: Mesenchymal stem cell
NIR: Near-infrared
NIR-I: Near-infrared region I
NIR-II: Near-infrared region II
NO: Nitric oxide
NPs: Nanoparticles
NSs: Nanosheets
*P. gingivalis*: *Porphyromonas gingivalis*
PB: Prussian blue
PC: Proanthocyanidins
PCE: Photothermal conversion efficiency
PCM: Phase-change materials
PDA: Polydopamine
PDT: Photodynamic therapy
PEG: Polyethylene glycol
PLGA: Poly lactic acid-co-glycolic acid
PLLA: Polylactic acid
POD: Photothermal and peroxidase
PTAs: Photothermal agents
PTT: Photothermal therapy
rGO: Reduced graphene oxide
RCT: Root canal therapy
RNS: Reactive nitrogen species
ROS: Reactive oxygen species
*S. aureus*: *Staphylococcus aureus*
*S. gordonii*: *Streptococcus gordonii*
*S. mutans*: *Streptococcus mutans*
*S. oralis*: *Streptococcus oralis*
*S. sanguinis*: *Streptococcus sanguinis*
*S. sobrinus*: *Streptococcus sobrinus*
SDT: Sonodynamic Therapy
SEM: Scanning electron microscopy
SNP: Sodium nitroprusside
SRP: Scaling and root planning
TC: Tetracycline
TRAP: Tartrate-resistant acid phosphatase
UBI: Ubiquicidine
VEGF: Vascular endothelial growth factor
